# Insights into the Aroma Profile of Sauce-Flavor Baijiu by GC-IMS Combined with Multivariate Statistical Analysis

**DOI:** 10.1155/2022/4614330

**Published:** 2022-03-29

**Authors:** Wenchao Cai, Yurong Wang, Wenping Wang, Na Shu, Qiangchuan Hou, Fengxian Tang, Chunhui Shan, Xinquan Yang, Zhuang Guo

**Affiliations:** ^1^Hubei Provincial Engineering and Technology Research Center for Food Ingredients, Hubei University of Arts and Sciences, Xiangyang, Hubei Province, China; ^2^School of Food Science, Shihezi University, Shihezi, Xinjiang Autonomous Region, China; ^3^Xiangyang Maotai-Flavor Baijiu Solid-State Fermentation Enterprise-University Joint Innovation Center, Xiangyang, Hubei Province, China; ^4^Xiangyang Maotai-Flavor Baijiu Solid-State Fermentation Key Laboratory, Xiangyang, Hubei Province, China

## Abstract

Aroma is among the principal quality indicators for evaluating Baijiu. The aroma profiles of sauce-flavor Baijiu produced by 10 different manufacturers were determined by GC-IMS. The results showed that GC-IMS could effectively separate the volatile compounds in Baijiu, and a total of 80 consensus volatile compounds were rapidly detected from all samples, among which 29 volatile compounds were identified, including 5 alcohols, 14 esters, 2 acids, 2 ketones, 5 aldehydes, and 1 furan. According to the differences in aroma profile found by multivariate statistical analysis, these sauce-flavor Baijiu produced by 10 different manufacturers can be further divided into three types. The relative odor activity value of the identified volatile compounds indicated that seven volatile compounds contributed most to the aroma of sauce-flavor Baijiu in order of aroma contribution rate, and they were ethyl hexanoate, ethyl pentanoate, ethyl 2-methylbutanoate, ethyl octanoate (also known as octanoic acid ethyl ester), ethyl 3-methylbutanoate, ethyl butanoate, and ethyl isobutyrate. Correspondingly, the main aromas of these sauce-flavor Baijiu produced by 10 different manufacturers were sweet, fruity, alcoholic, etheral, cognac, rummy, and winey. On the one hand, this study proved that GC-IMS is well adapted to the detection of characteristic volatile aroma compounds and trace compounds in Baijiu, which is of positive significance for improving the aroma fingerprint and database of sauce-flavor Baijiu. On the other hand, it also enriched our knowledge of Baijiu and provided references for the evaluation and regulation of the flavor quality of sauce-flavor Baijiu.

## 1. Introduction

Baijiu is a distilled liquor that originated in China, which is famous for its ancient brewing process as well as unique flavor and is one of the seven most famous distilled liquors in the world. Meanwhile, Baijiu is also the most consumed liquor in China, with an annual production of over 78 billion tons [1]. Baijiu has formed its unique brewing technology and style during the long development process. Baijiu is generally made from cereals or sorghum, with Daqu, Xiaoqu, or Fuqu as saccharifying starters, through steaming, stirring, solid-state fermentation, solid-state distillation, storage, and blending [2]. Owing to the influence of raw materials, climate, geography, environment, and differences in the brewing process, as well as diverse style characteristics, Baijiu can be classified into many types, such as strong-flavor, light-flavor, sauce-flavor, and sesame-flavor [1, 3, 4]. Among them, sauce-flavor Baijiu (SFB) is strongly favored by consumers for its characteristic of mellow taste and long aftertaste. The sensory characteristics of SFB are slightly yellowish and transparent, prominent soy sauce aroma, delicate fragrance, pleasantly strong, and full liquor body as well as a lingering aroma in empty glass [5]. Owing to the unique production technology of “high temperature Daqu making, high temperature stacking, high temperature fermentation, high temperature distillation, and multiple rounds of fermentation,” SFB has the features of numerous volatile compounds and complex aroma [6, 7]. The core source of the solid-state fermentation of traditional SFB, derived from the complex microorganisms involved in the fermentation, and its fermentation pattern belongs to a bilateral fermentation with simultaneous saccharification and fermentation, which is different from other distilled liquors produced by unilateral fermentation with only added starter [8, 9]. The abundant microorganisms and their metabolically produced enzyme systems form and enrich a large number of liquor aroma compounds in a unique and suitable brewing environment and technology, which endows SFB with the unique style and quality, although these volatile aroma compounds account for only 1–2% (v/v) of the whole Baijiu [4, 8].

Research on the main components of volatile aroma compounds in SFB has been a focus in the field of Baijiu [2, 10]. Electronic nose, gas chromatography mass spectrometry (GC-MS), and gas chromatography olfactometry (GC-O) are the most common methods for the evaluation of aroma in foods. Electronic nose is simple to operate and reproducible, but it has low precision, sensors drift easily, and unable to qualitatively identify individual volatile compounds.

Electronic nose has simple operation and good reproducibility; however, its precision is low, sensors are easy to drift and unable to achieve qualitative analysis of individual volatile compounds; GC-MS suffers from several disadvantages, such as complicated preprocessing procedure and difficult to resolve fragmented peaks; while GC-O lacks of reproducibility, stability, and objectivity [11, 12]. Ion mobility spectrometry (IMS) is a detection technique developed in the late 1960s to detect a wide range of different chemicals by the mobility of gaseous ions in a uniform electric field. IMS works under ambient pressure conditions with low detection limit for single compounds, and the mobility is only related to the compound itself; therefore, its qualitative analysis is accurate and particularly suitable for trace detection of volatile compounds, but IMS has a low resolution for mixtures as well as compounds with similar mobility [13]. The combination of gas chromatography (GC) and IMS can well solve the problem of low resolution of IMS, which greatly broadens the application of traditional IMS.

Gas chromatography ion mobility spectrometry (GC-IMS) is an emerging technique for rapid detection of volatile compounds in samples using GC coupled with IMS [14]. This technique is based on the initial separation of complex compounds by the capillary column of GC, followed by the chemical ionization reactions that occur by the addition of individual compounds into the ionization reaction zone of the IMS, and achieved by comparing the varying drift times required for ionized compounds to pass a fixed distance (drift tube) in a specific electric field through required for different drift times [15, 16]. GC-IMS is rapid, sensitive, and easy to operate, it not only significantly improves the signal response quality of IMS but also enriches the chemical information obtained after GC separation, fully exerting the advantages of each of the two techniques. Compared with the GC-MS method, the GC-IMS technique has a low detection limit, and the sample volatile compounds need no pretreatment such as enrichment and concentration, which can be directly headspace injected, and all volatile compounds can be analyzed more realistically and comprehensively, while also avoiding the disadvantages such as complicated operation and difficult fragment ion dissociation spectra [12]. Taylor et al. [15] found that although both techniques of GC-MS and GC-IMS can distinguish samples, GC-IMS is markedly less costly and requiring only about 20 min for one sample analysis. Because IMS has high-response sensitivity to compounds with high electronegativity or strong proton affinity, and many volatile aroma compounds in food have high electronegativity or strong proton affinity functional group structures, such as amino, sulfhydryl, halogen groups as well as organic compounds, and aromatic compounds including amino, sulfhydryl, and halogen groups, aromatic compounds as well as organic compounds such as aldehydes, ketones, and ethers containing unsaturated bond structures [12]. For this reason, it is a unique advantage to employ GC-IMS for the analysis of food aroma profile. GC-IMS is currently widely used in the evaluation of aroma profile of olive oil [16, 17], ham [18], honey [19, 20], and *Tricholoma matsutake* Singer [21, 22]. However, to the best of our knowledge, the application of GC-IMS for the detection of volatile aroma compounds in SFB has not been reported yet.

In this study, the commercially available premium SFB produced by 10 different manufacturers were used to determine the aroma profiles and characteristics of the samples by GC-IMS; meanwhile, multivariate statistical analysis method was applied to explore the regularity and potential types of volatile compounds in different SFB, in the hope of providing new methods and ideas for the quality evaluation of SFB.

## 2. Materials and Methods

### 2.1. Sample Collection

The following 10 premium sauce-flavor Baijiu (MT, JS, LM, GT, XJ, DYT, ZJ, LX, JS, and YZH) from 10 different distilleries were under investigation and coded from A to J, respectively. All these 10 representative Baijiu samples (53% ethanol by volume, 500 mL for each) were bottled in 2019, supplied by corresponding distilleries and stored at ambient temperature and under light free conditions.

### 2.2. Sample Pretreatment

A quantity of 100 *μ*L of each SFB sample was diluted with 900 *μ*L of distilled water and put into a 20 mL headspace sampling vial and then sealed with magnetic cap and silicone septum for later use.

### 2.3. Determination of GC-IMS

GC-IMS analysis was carried out on a commercial GC-IMS (FlavourSpec@, Gesellschaft für Analytische Sensorysteme GmbH Inc, Dortmund, Germany), equipped with an automatic headspace sampling unit (CTC-PAL RSI, CTC Analytics AG, Zwingen, Switzerland), a 2.5 mL Hamilton syringe to improve the reproducibility of measurements. All parameters were set in the light of Arroyo-Manzanares et al. [18] and Guo et al. [22] with slight modifications according to practical situations.

#### 2.3.1. Automatic Headspace Sampling Unit Parameters

The sealed headspace sampling vials were placed into the automatic headspace sampling unit with automatic headspace injection method and incubated (500 r/min) at 60°C for 10 min. Afterward, 100 *μ*L of the sample in the headspace sampling vial was injected into the GC-IMS at a syringe temperature of 85°C.

#### 2.3.2. GC Parameters

The GC was equipped with a WAX 30 m ID: 0.53 mm capillary column, and set the GC column temperature to 60°C. The carrier gas during the assay was high-purity N_2_. The carrier gas flow rate was set as follows: the initial flow rate was 2 mL/min hold for 10 min, then increased linearly to 10 mL/min hold for 10 min, and finally increased linearly to 100 mL/min hold for 10 min. The total analysis time was 30 min.

#### 2.3.3. IMS Parameters

The IMS was equipped with a drift tube of 98 mm long. Linear voltage inside the drift tube was 500 V/cm. The temperature of IMS drift tube was set to 45°C, the drift gas during the assay was high-purity N_2_, and the flow rate of the drift gas was kept constant at 150 mL/min. The ionization source inside the drift tube was deuterium radiation, namely, beta ray, ^3^H was the radioactive source, and the ionization mode was in positive ion mode.

All the samples were twice determined in triplicate experiments. GC/IMS data consist of the signal intensity, the drift time of the ions in the IMS drift tube, and the retention time in the GC column and the signal intensity. Data were processed using the instrument's home-contained analysis software Laboratory Analytical Viewer (LAV). Identification of volatile compounds was achieved by comparing the retention index and the relative drift time of samples with those of the qualitative software GC-IMS Library Search (built-in NIST2014 database and IMS database). Since GC-IMS did not respond to small molecule n-alkanes, the retention indexes were calculated by using an *n*-ketones series (C4–C9) under the same chromatographic conditions as the samples. The plug-in Reporter in LAV was used to perform sample GC-IMS spectra (two-dimensional top view, three-dimensional spectrum, and difference spectrum) contrast, and the plug-in Gallery plot was used to perform GC-IMS fingerprints contrast.

### 2.4. Calculation of Relative Odor Activity Value

With reference to the description of Zhu et al. [23] and Wei et al. [24], relative odor activity value (ROAV) was adopted to evaluate the contribution of identified individual volatile compounds to the aroma. On the basis of the reports in the literature, the ROAVmax of the volatile compound that contributed most to the sample aroma as 100 was defined, and then the ROAV of the other volatile compounds (*A*) was calculated as follows:

(1) $$\begin{array}{c} \text{ROAV}\approx \frac{C\%A}{C\%\max}\times \frac{T\max}{\text{TA}}\times 100. \end{array}$$

In the formula, *C*%*A*, TA were the relative contents and corresponding odor thresholds of each volatile compound, and *C*%max *T*max were the relative contents and odor thresholds of the volatile compound that contributed the most to the aroma of the sample, respectively.

### 2.5. Statistical Analysis

The volatile compounds identified by the home-contained software of GC-IMS were conducted multivariate statistical analysis methods (cluster analysis, multivariate analysis of variance, principal component analysis, linear discriminant analysis effect size, and analysis of variable importance in projection).

Cluster analysis (CA), multivariate analysis of variance (MANOVA), principal component analysis (PCA) and analysis of variable importance in projection (VIP) were performed using *R* software (version 4.0.2, Murray Hill, New Jersey, USA). While linear discriminant analysis effect size (LEfSe) was performed via the online interface utilizing the Huttenhower Lab Galaxy Server (https://huttenhower.sph.harvard.edu/galaxy/). The figures were plotted by Origin (version 2019, Origin Lab, Hampton, Massachusetts, USA) and *R* software.

## 3. Results

### 3.1. Comparative Analysis of GC-IMS Spectrum

Figure. S1 is a three-dimensional GC-IMS spectrum (retention time, drift time and peak intensity) of aroma profiles in the SFB samples produced by 10 different manufacturers according to LAV analysis. It is known from Figure S1 that the retention time of volatile compounds during ionic drift is directly proportional to the quadratic of the drift time, that is, the longer the retention time of volatile compounds in drift spectrum, the longer their corresponding drift time is. The color change of the volatile compounds that underwent drift when retained from 500 to 550 s was significant, indicating that the volatile compounds retained until the final drift out differed more among different SFB samples. The vast majority of volatile compounds have been effectively drifted at 200 ± 50 s, which also reflects that volatile compounds in SFB samples were well separated by GC-IMS. For observation convenience, the GC-IMS spectrum top view was taken below for differential contrast.

**Figure 1:**
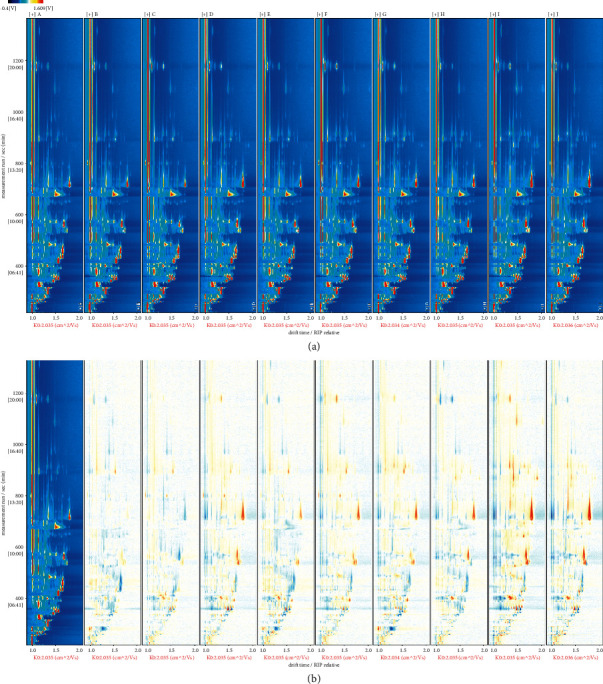
Two-dimensional top view of GC-IMS spectrum.

**Figure 2:**
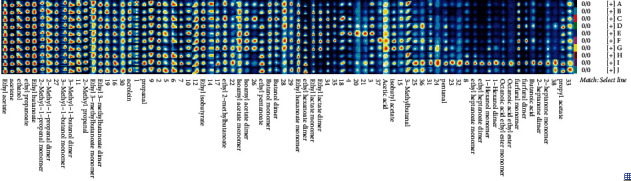
Aroma fingerprint of SFB samples produced by 10 different manufacturers generated using gallery plot plugin of LAV software.

Figure 1 is a two-dimensional top view of Figure. S1 in which the background of the map in Figure 1(a) is blue, and the redder the color is, the higher content of this compound. It is noted that the first red vertical bar at the early ionic drift time of 1.0 ms in GC-IMS is the reactive ion peak (RIP) of water (Figure 1(a)), which is formed because the headspace injection gas contains water vapor, and the energy electrons generated from the ionization source of radioactive tritium (^3^H) of IMS undergo a gas-phase reaction, allowing protonated water molecules and hydrogen ions to aggregate to form water molecules. When volatile compounds are present in the ionization region of the IMS, the RIP intensity of water will decrease or disappears [25]. The drift time of the GC-IMS spectrum was normalized with RIP to avoid the change in ion drift time due to temperature and pressure biases during detection [26]. Because of the divergences in the ion mass and the number of charges of the volatile compounds, the different ions were separated under the collision effect of neutral gas molecules in the ion drift zone of the device and the applied electric field, which can qualitatively and quantitatively analyze the components of volatile compounds by combining the ion drift time with the intensity of the ion reaction peak [27]. As can be intuitively seen in Figure 1(a), the volatile compounds and aroma profiles of the SFB samples produced from 10 different manufacturers had a high similarity, since all the samples in this study were of the same flavor type. To better compare the discrepancies of volatile compounds among SFB samples produced from different manufacturers, a differential contrast mode was adopted for Figure 1(b): The spectrum of sample A in Figure 1(b) was selected as the reference, and the spectra of other samples were compared with the reference. If the contents of volatile compounds in the two are consistent, the background is white after subtraction, whereas red represents the compound at a higher content than the reference, and blue stands for the compound at a lower content than the reference. Interestingly, a volatile compound may produce multiple rather than one signal spot (monomer and dimer or even multimer), depending on the content of the compound [18]. It can be seen that there are still differences in the contents of volatile compounds among different SFB samples, which are reflected in the location, number, and corresponding peak intensities. Without qualitative analysis of the compounds, the specific information of each volatile compounds in Figure 1 cannot be determined. Hence, all the compound peaks were selected as points to be analyzed by the Gallery plot plugin of LAV software to generate the aroma fingerprint (Figure 2).

### 3.2. Identification of Volatile Compounds

The distinguishable differences among the SFB samples produced by 10 different manufacturers can be seen from Figure 2, especially for the sample I. Using LAV software, 80 characteristic peaks were separated in all SFB samples, and 29 volatile compounds (see Table S1 for details) were qualitatively identified against the NIST2014 gas-phase retention index database as well as the IMS database. There were 12 compounds which, owing to their high content, not only existed as monomers but also generated dimers when separated (Figure 2). Same as the conclusion in Figures 1 and 2 depicts that the aroma profiles of SFB samples produced by different manufacturers were similar, but there were differences in the contents of specific volatile compounds, and some volatiles appeared as proton bonded monomers or dimers [28]. Moreover, the volatile compounds on the right of the gallery plot were more abundant among the different SFB samples, whereas those on the left were relatively less abundant. In addition, propyl acetate, 2-heptanone, butanoic acid, octanoic acid, 1-hexanol, ethyl heptanoate on the right side of the gallery plot, had the highest content in sample I, which may cause its aroma profile to differ from other SFB samples.

**Figure 3:**
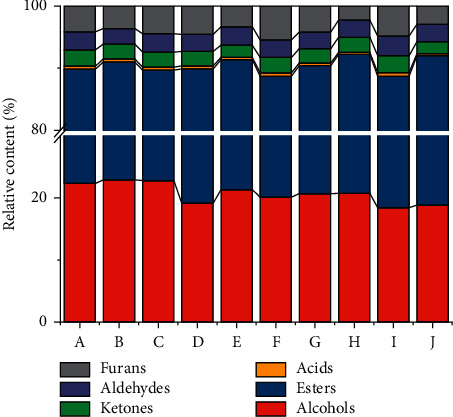
Relative content of volatile compounds in SFB samples produced by 10 different manufacturers.

Twenty-nine volatile compounds with carbon chains all within C2–C10 were identified in the SFB samples and could be classified into 6 classes, including 5 alcohols (coded A1 to A5), 14 esters (coded B1 to B5), 2 acids (coded C1 to C5), 2 ketones (coded D1 to D5), 6 aldehydes (coded E1 to E5), and 1 furan (coded F1) (Table S1). The relative content of different classes of volatile compounds in SFB samples produced by 10 different manufacturers is presented in Figure 3. The relative content of esters in all SFB samples was the highest with an average relative content accounted for 69.34% of the total contents of volatile compounds, followed by alcohols, which accounted for 20.98%, and both of which together accounted for 90.32% of the volatile compounds identified. Other classes of volatile compounds accounted for a lower relatively low total contents of volatile compounds, with acids accounting for 0.46%, ketones for 2.35%, aldehydes for 2.84%, and furans for 4.04%. It follows that esters and alcohols are the two most dominant classes of volatile compounds in SFB samples, which have also been consistently considered as the skeleton compounds that contribute the most to the aroma of the SFB [29]. Among all the SFB samples, the highest content of alcohols was found in sample A, B, and C, whereas the highest content of esters was in sample D, H, I, and J.

### 3.3. Multivariate Statistical Analysis

#### 3.3.1. CA

**Figure 4:**
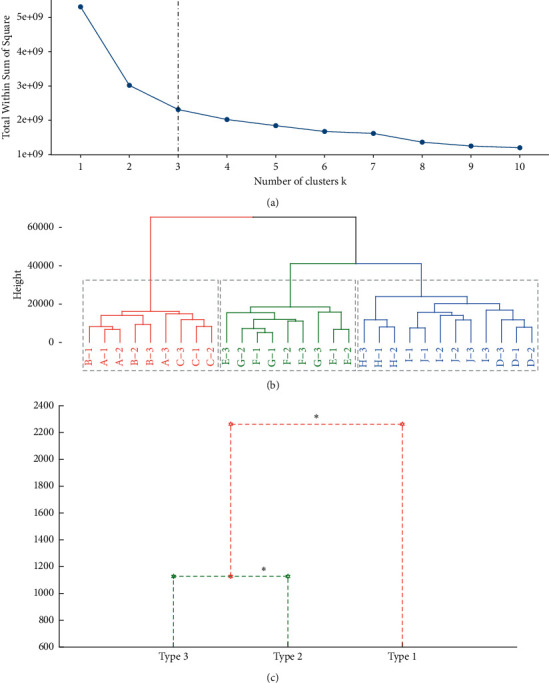
Optimal number of clusters based on the aroma profiles of SFB samples calculated by *k*-means algorithm (a); dendrogram based on UPGMA (b); dendrogram based on Bray–Curtis distance calculated using Mahalanobis distances as well as MANOVA (c).

In order to explore the regularity of aroma profiles in different SFB, GC-IMS characteristic peak intensities were selected as the feature variables, and *K*-means clustering algorithm was performed [30]. As it is observed in Figure 4(a), the total within sum of square decreased sharply at the numbers of clusters 1 and 2 and leveled off until 3. Subsequently, the clustering according to the aroma profiles of the SFB samples was visualized using an unconstrained CA based on the unweighted pair group method with arithmetic mean (UPGMA) Figure 4(b) [31]. It was found that at the height of 30,000, SFB samples were divided into three clusters, indicating that there are significant aroma differences among sample groupings when the samples were sorted into *k* = 3 clusters. There into, cluster 1 comprises Baijiu sample A, B, and C, cluster 2 mainly consists of sample E, F, and G, while cluster 3 mainly includes sample D, H, I, and J. A constrained CA based on Mahalanobis distances combined with MANOVA (Figure 4(c)) further proved significant differences (*p* < 0.05) among the aroma profiles of SFB samples in the three clusters [32]. As a result, the SFB samples of these three clusters were defined here as type 1, type 2, and type 3, respectively. Besides, combining Figure 4(b) with Figure 4(c) leads to the suggestion that the aroma profile of type 1 SFB is more different from the other two types.

#### 3.3.2. PCA

**Figure 5:**
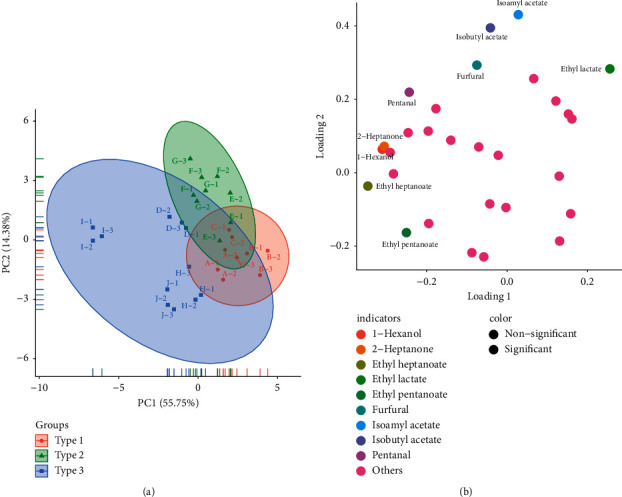
PCA loading plot (a) and score plot (b) based on the aroma profiles of SFB samples.

According to the aroma profile of SFB samples, PCA, an unconstrained analysis with ellipse confidence was conducted to dimensionally reduce the feature vectors to eliminate data redundancy so as to observe the aroma profiles of different SFB samples more intuitively (Figure 5). The first two principal components (PC), PC1 and PC2, explained 70.13% of the total variance, accounting for 55.75%, and 14.38% of the variance, respectively. As shown in PCA score plot (Figure 5(a)), all SFB samples could be well differentiated into three clusters according to the differences in the aroma profile, and the clustering results were in line with the assertion of CA (Figures 4(b) and 4(c)). Notably, the spatial distribution of the sample I was more distant from the other SFB samples, which resulted from propyl acetate, 2-heptanone, butanoic acid, octanoic acid, 1-hexanol, and ethyl heptanoate in the aforementioned gallery plot (Figure 2). Nine volatile compounds with the largest spatial distribution distances were highlighted in the PCA loading plot (Figure 5(b)), presumably that they led to the further division of SFB into three types. A combination of the PCA score plot (Figure 5(a)) and loading plot (Figure 5(b)) reveals that the type 2 SFB samples, ethyl lactate, isoamyl acetate, and isobutyl acetate were all distributed in the top right of the PCA plot, implying that the type 2 SFB samples were enriched with these three esters. Meanwhile, type 3 SFB samples, ethyl pentanoate, ethyl heptanoate, 1-hexanol, and 2-heptanone were all located on the left side of PCA plot, which illustrated higher contents of these four volatile compounds in type 3 SFB samples.

#### 3.3.3. Identification of Aroma Markers

**Figure 6:**
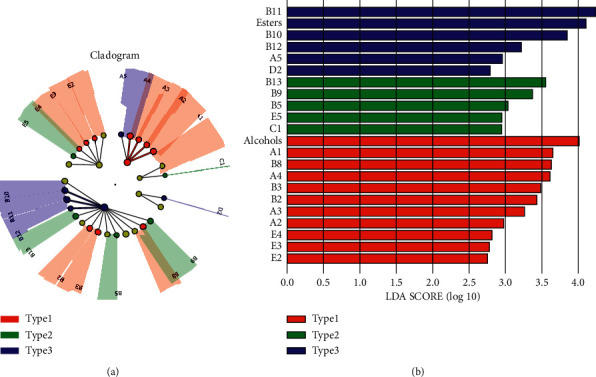
Identification of differential volatile compounds among different types of SFB samples via LEfSe. Cladogram of the volatile compounds (a). Significant differential volatile compounds of type 1 SFB samples, type 2 SFB samples and type 3 SFB samples are represented by red, green and blue, respectively. while other compounds are represented by yellow. Branch areas are shaded according to the highest-ranked variety for that taxon. The LDA score indicates the level of differentiation among different types of SFB. A threshold value of 2.0 was used as the cut-off level. Horizontal bar chart showing differential volatile compounds (b). Significant differential volatile compounds of type 1 SFB samples, type 2 SFB samples and type 3 SFB samples are represented by red, green and blue, respectively.

To quantitatively evaluate the extent to which the volatile compounds with significant differences (*p* < 0.05) among different types of SFB affected the classification, as well as to clarify the aroma characteristics of different types of SFB, LEfSe was conducted (Figure 6) [33]. By Kruskal–Wallis rank sum test, a total of 20 differential volatile compounds with significantly different contents (*p* < 0.05) were obtained among all three types of SFB. Among them, the relative contents of alcohols, A1 (ethanol), B8 (ethyl 3-methylbutanoate), A4 (3-methyl-1-butanol), B3 (ethyl isobutyrate), B2 (ethyl propanoate), A3 (butanol), A2 (2-methyl-1-propanol), E4 (3-methylbutanal), E3 (acrolein), and E2 (2-methyl propanal) were significantly more abundant (*p* < 0.05) in type 1 SFB samples than in the other two types of SFB, B13 (ethyl lactate), B9 (isoamyl acetate), B5 (isobutyl acetate), E5 (pentanal), and C1 (acetic acid) were significantly higher (*p* < 0.05) in type 2 SFB samples, whereas B11 (ethyl hexanoate), esters, B10 (ethyl pentanoate), B12 (ethyl heptanoate), A5 (1-hexanol), and D2 (2-heptanone) were significantly more abundant in type 3 SFB samples. This is also in general agreement with the results shown in Figure 3 and PCA. Moreover, the number of differential volatile compounds in type 1 SFB samples is more, which may explain the greater divergence of type 1 SFB from the other two types in CA (Figures 4(b) and 4(c)).

Taking linear discriminant analysis (LDA) score of 3.5 as cutline, volatile compound with LDA score greater than 3.5 in each types of SFB were defined as aroma maker, which is the characteristic compound for this type of SFB. The aroma markers were alcohols, A1 (ethanol), B8 (ethyl 3-methylbutanoate), and A4 (3-methyl-1-butanol) in type 1 SFB samples, B13 (ethyl lactate) in type 2 SFB samples, whereas B11 (ethyl hexanoate) and esters in type 3 SFB samples.

#### 3.3.4. Identification of Aroma-Active Compounds

Olfactory sensitivity to different volatile compounds varies greatly due to differences in chemical composition, molecular structure, along with specific binding degree of compounds to the olfactory receptor cells in animal nasal passages, and the lowest concentration at which a person can sense a volatile compound is generally referred to as the “odor threshold” [34]. The perception of aroma depends on the content and threshold of volatile compounds, the conclusion obtained by considering only any one of them is incomprehensive, or even wrong; an objective evaluation can only be made by combining the two, which means the indicators odor activity value (OAV) or ROAV, representing the ratio of the content of the volatile compound to its odor threshold [34, 35].

To gain insight into the volatile compounds in SFB samples, ROAV was calculated to identify key volatile compounds in SFB to evaluate the contribution of these compounds to the SFB aroma [23, 24]. The higher the ROAV of a volatile compound is, the more significantly it contributes to the aroma of SFB [7]. The odor thresholds of the identified volatile compounds in the SFB samples were obtained by consulting previous reports, and their ROAV were calculated [1, 7, 36–39]. In the light of Niu et al. [40], ethyl hexanoate is the most dominant aroma-active compounds in SFB. Therefore, the ROAV of ethyl hexanoate was defined as 100, and the ROAV of other volatile compounds were calculated by the formula in Section 2.3, the results are shown in Table S2. In all SFB samples, there were 10 volatile compounds with ROAV greater than 8, whereas ROAV for the remaining volatile compounds was less than 0.6.

**Figure 7:**
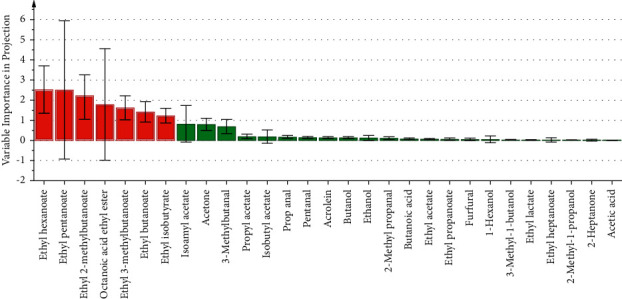
Aroma-active compounds identified by ROAV and VIP. Volatile compounds with VIP > 1 and volatile compounds with VIP < 1 are represented by red and green, respectively.

VIP can quantify the contribution of each variable to a sample, and usually variables with VIP greater than 1 are considered as important variables in a sample. ROAV combined with VIP (Figure 7) screened out the seven volatile compounds that contributed most to the aroma of SFB, which were sequentially ranked by the contribution: ethyl hexanoate, ethyl pentanoate, ethyl 2-methylbutanoate, ethyl octanoate, ethyl 3-methylbutanoate, ethyl butanoate, and ethyl isobutyrate.

## 4. Discussion

The aroma profile is one of the most crucial characteristics of distilled liquors, and it is affected by multiple factors, especially raw materials, climate, environment, process, and so on [7, 41, 42], and the above factors all differ according to manufacturers. For this reason, it is necessary to explore the similarities and differences between aroma profiles of the Baijiu with the same flavor-type produced by different manufacturers.

At present, a common assay for the determination of volatile compounds in samples is the use of GC-MS. The GC-IMS technique combines the separation ability of GC and the high sensitivity of IMS, which has the advantages of simple operation, nondestructive detection, and good repeatability. Moreover, it requires no sample pretreatment and the analyzed temperature is much lower than that of GC-MS. Thus, the GC-IMS can more accurately reflect the volatile compounds of samples [14, 18]. In this study, GC-IMS was utilized to determine the volatile compounds of SFB samples produced by 10 different manufacturers, and detected 80 volatile compounds that existed in all SFB samples (Figure 2). This is because, as Baijiu of the same flavor-type, these samples have similar aroma profiles, and the 80 volatile compounds are probably the skeleton compounds in SFB. As shown in Figure 2, both monomer and dimer are present for 2-methyl-1-propanol, butanol, 3-methyl-1-butanol, 1-hexanol, ethyl 3-methylbutanoate, isoamyl acetate, ethyl hexanoate, ethyl heptanoate, ethyl lactate, ethyl octanoate, 2-heptanone, and furfural. The retention time of monomer and dimer of the same compound are similar, but the drift time are different, which is related to the content of the compound, and high content compounds can promote the combination of protonated molecules as well as neutral molecules in the ionizing region to generate dimers [18, 21, 26, 43]. In addition, Lantsuzskaya et al. [44] and Lantsuzskaya et al. [45] found that the formation of dimer is also related to the high-proton affinity of the analytes, since the proton affinity of some compounds is higher than that of the water, the protons of the reactants are transferred to the high-affinity proton affinities, resulting in the formation of dimers or polymers. The newly generated dimers are larger in mass than the monomers, so they drift at lower rate and with higher drift time, leading to the appearance of multiple signals in the ion mobility spectrum for some compounds during the assay, whereas double data of monomers and dimers can identify the compounds more precisely.

Based on the aroma profile of the samples, multivariate statistical analysis divided the SFB into three distinct types (Figures 4 and 5). LEfSe further pinpointed the aroma markers in different types of SFB (Figure 6), from which different types of SFB distinguished, and these various contents of aroma markers constituted the flavor unique to each type of SFB.

Most distilled liquors worldwide are characterized by alcohol aroma, whereas the overall aroma of Baijiu is dominated by ester aroma, which is also a major feature of Baijiu, and SFB is no exception [46, 47]. Esters are generally formed by esterification of alcohols and acids, especially ethanol, during Baijiu fermentation and aging, and mainly endow the liquor with rich fruity aroma characteristics [4, 7, 40, 48]. The amount and contents of esters in SFB samples were considerably higher than those in the other classes of volatile compounds, which dominated (Figure 3, Table S1). Among the 14 esters, higher amounts were detected in ethyl hexanoate, ethyl acetate, ethyl butanoate, ethyl 3-methylbutanoate along with ethyl pentanoate (Figure 2, Table S1), all of which were ethyl esters, and the results were similar to those reported in SFB [1, 2, 40]. The ethyl esters are were the most important yeast-synthesized volatile compounds during Baijiu fermentation, presenting pleasant fruity aromas [41]. Furthermore, the importance of ethyl esters was also demonstrated by the presence of ethyl esters separately in both aroma markers of three types of SFB, in which, ethyl 3-methylbutanoate in type 1 SFB, ethyl lactate in type 2 SFB, and ethyl hexanoate in type 3 SFB. ethyl 3-methylbutanoate with a berry, pineapple and fruity aroma is rarely reported in SFB, but has been proved to have high content with high aroma contribution in light-flavor Baijiu [49], strong-flavor Baijiu [50], qingke Baijiu [37], sesame-flavor Baijiu [51, 52], chixiang-flavor Baijiu [53], and laobaigan-flavor Baijiu [39]. Ethyl lactate, which is generally secreted by lactic acid bacteria, is essential for stabilizing Baijiu flavor and exhibits high stability during Baijiu aging [48]. As for ethyl hexanoate, which has a sweet, fruity, pineapple, green banana-like aroma, is regarded as the most important and indispensable skeleton compounds in Baijiu [1, 2, 7, 8, 40, 41].

Alcohols not only present fruity, green and alcoholic aroma, but also have a sweet alcoholic taste [7]. Noguerol-Pato et al. [54] found that when the concentration of alcohols was lower than 300 mg/L, it could make the flavor of alcoholic beverages to achieve the desired complexity. Most of alcohols were formed from deaminization reaction of amino acids in anaerobic environment or from decarboxylation reaction of sugars in aerobic environment by yeast [7]. Besides, a small number of alcohols could also be produced by yeast via chemical reduction in the corresponding aldehydes [40]. Among the alcohols, two saturated and unsaturated alcohols were detected, respectively, in addition to ethanol. The alcohol with the highest content was 3-methyl-1-butanol, followed by ethanol (Figure 2, Table S1). Niu et al. [40] and Fan et al. [2] also proved that 3-methyl-1-butanol was the most abundant alcohol in SFB. 3-methyl-1-butanol, this branched-chain saturated fatty alcohol formed by the deamination and decarboxylation reactions of amino acids is one of the skeleton compounds of the flavor composition in alcoholic beverages, with a malt whiskey and burned aroma, which can make the flavor rich and full, meanwhile, it is also the precursor to form esters [4, 55–57]. As one of the aroma markers in type 1 SFB, it has made a positive contribution to the aroma.

Acids have fruity, cheese, fatty and rancid aromas, mainly affect the taste and aftertaste of Baijiu, enhance aroma and fragrance as well as reduce the stimulation [50]. Meanwhile, acids in Baijiu have also been proved to help improve the color stability and antioxidant capacity of final products [48]. Two short-chain acids were identified from SFB samples, namely acetic acid and butanoic acid. Acids are the precursors of esters, among them, the contribution of short-chain acids, such as acetic acid with vinegar aroma and butanoic acid with cheesy aroma, to Baijiu flavor focuses on mouthfeel, and moderate content of short-chain acids has the effect of balancing liquor taste and coordinating aroma, while they will inhibit and cover other volatile compounds in Baijiu when the content is too high [7, 36, 40, 56]. Consequently, the ability of GC-IMS to rapidly and accurately detect short-chain acids is of positive significance to Baijiu.

Carbonyl compounds (ketones and aldehydes) are formed from unsaturated fatty acids and amino acids under oxygen conditions [4]. Two ketones were identified, namely 2-heptanone and acetone. Noguerol-Pato, et al. [58] believed that 2-heptanone may be associated with deterioration of raw materials and will bring unpleasant off-flavors to the consumer. Acetone has a sweet, fruity, and etherous aroma, and its presence has not been previously reported in SFB. However, in the present study, GC-IMS not only captured its presence in every sample, but also in combination with ROAV demonstrated that it also had an appreciable impact on the aroma of SFB (Figure 7, Table S1). The chain length of aldehydes is closely related to the odor threshold and aroma characteristics, low molecular weight (<150 DA) aldehydes tend to produce unpleasant aromas [59], whereas high molecular weight aldehydes have sweet and fruity aromas [60]. A total of six short-chain aldehydes were identified in SFB samples. Short chain (C3–C5) aldehydes in Baijiu were usually derived from amino acid degradation or autoxidation of oils and fats [61, 62]. Straight-chain aldehydes (propanal, pentanal) and branched-chain aldehydes (2-methyl propanal, 3-methylbutanal) generally provide green grassy and pungent aromas, while unsaturated aldehydes (acrolein, furfural) are associated with vegetable and fishy smell [63].

Furfural, contributing to sweet and almond-like aroma that is the most important furans in SFB and is even a marker compound of SFB [2, 7]. It was produced by the fragmentation or cyclization of 3-deoxyacetone as a highly reactive intermediate during nonenzymatic browning reactions such as Maillard reaction (involving Amadori rearrangement compounds) and pyrosaccharification (involving sugar degradation) [4].

Pyrazines are ubiquitous, and one of the characteristic volatile compounds in SFB, which are generally produced by the Maillard reaction during the high-temperature Daqu-making and high-temperature fermentation of SFB or the reaction of acetoin and free ammonia produced by microbial metabolism, they are also one of the sources of the typical roasted and nutty aroma in SFB [7, 41, 64]. Regrettably, GC-IMS did not identify pyrazines in the sample.

Although GC-IMS can effectively identify volatile compounds, it does not reflect the importance of volatile compounds to aroma. Therefore, GC-IMS and ROAV were applied in this study to analyze the volatile compounds in SFB samples to evaluate their importance. The seven ethyl esters, including ethyl hexanoate, ethyl pentanoate, ethyl 2-methylbutanoate, ethyl octanoate, ethyl 3-methylbutanoate, ethyl butanoate, and ethyl isobutyrate were identified as aroma-active compounds in SFB. Correspondingly, the main aromas of all SFB samples are mainly in two categories, one is the sweet fruity aromas generally possessed by ethyl esters, and the other is the alcoholic, etheral, cognac, rummy, and winey aromas to which ethyl butanoate, ethyl octanoate, and ethyl isobutyrate contributed [7, 36, 38]. Previously, Niu et al. [1] used GC to connect selective ion monitoring (SIM) mass spectrometry and identified six aroma-active compounds from SFB, comprising ethyl isobutyrate, ethyl butanoate, ethyl 2-methylbutanoate, ethyl isovalerate, ethyl pentanoate, and ethyl hexanoate. Niu et al. [40] employed GC-O combined with aroma extract dilution analysis to discover that ethyl butanoate, ethyl pentanoate, ethyl hexanoate, and ethyl octanoate are the aroma active compounds in SFB. These results are basically consistent with this study, signifying that GC-IMS combined with ROAV could effectively evaluate and identify aroma-active compounds in Baijiu.

It is worth mentioning that 1 ketones (acetone) and 3 aldehydes (propanal, acrolein, and pentanal) were first reported in SFB, all of them being low carbon chain and low retention index (RI) compounds. Furthermore, in the present study, a total of 29 volatile compounds whose carbon chains were all within C2 to C10 were identified from SFB samples via GC-IMS, consisting of 20 compounds with RI <1200 (accounted for 68.97%), and 26 compounds with RI <1500 (accounted for 89.66%) (Table S1). Whereas most previous studies of SFB aroma were analyzed by GC-MS, the carbon chains of detected volatile compounds were generally within C10 to C5 and showed low sensitivity for small molecular weight, low RI and trace compounds [2, 40]. There are some differences in the analytical results between the two techniques, most of them detected by GC-MS are large molecules (C10–C15) of volatile compounds with high RI and high contents, whereas GC-IMS can effectively capture the small molecules (C2 to C11) volatile compounds with low RI and low contents. This is mainly because, on the one hand, the detection sensitivity of GC-IMS is higher compared to GC-MS, especially for compounds with highly electronegative or high-proton affinity functional groups (amino, sulfhydryl, halogen groups along with organic compounds such as aldehydes, ketones, ethers containing unsaturated bond structures); on the other hand, relative to GC-MS, GC-IMS adopts a constant temperature rising flow mode with lower incubation temperature, injection temperature, as well as column temperature, and during the ascending flow process, the volatile compounds could be effectively captured and detected by IMS after preseparation by GC. Hence, GC-IMS were more precise for the detection of small molecular compounds. From the detection results, GC-IMS could be a good complement to GC-MS technique, which expanded the detection range of volatile compounds and reflected the aroma profile of Baijiu more comprehensively. However, due to the late start of GC-IMS, its database in the field of Baijiu is not well established, the qualitative results were available for only 29 of the 80 volatile compounds, and the remaining volatile compounds await further investigation. This is a common limitation of GC-IMS in the analysis of food volatile compounds, and it also illustrates that GC-IMS has great potential for development in the field of food aroma analysis [65]. Standards for acetone, propanal, acrolein, pentanal, and other unidentifiable volatile compounds in SFB will be adopted in the follow-up study to validate their roles.

## 5. Conclusion

In this study, GC-IMS combined with multivariate statistical analysis can achieve rapid and accurate detection of volatile compounds in Baijiu, and visualize the data to more intuitively exhibit the variations among samples. According to the GC-IMS database, 29 volatile compounds were identified in the SFB samples produced by different manufacturers, and acetone, propanal, acrolein, and pentanal were reported for the first time in SFB. SFB can be subdivided into three types, each with its own unique aroma marker. GC-IMS combined with ROAV could also effectively evaluate and identify aroma-active compounds in Baijiu. But because of the late start of GC-IMS and the IMS database is not well established, some of the volatile compounds could not be qualitatively identified. In the subsequent studies, the unidentified volatile compounds will be subsequently verified with standards or other means, enriching the database of volatile compounds in Baijiu field.

This study demonstrates that GC-IMS can well compensate for the deficiencies of GC-MS in detection, expand the detection range of volatile compounds in samples, and have a great application potential in the analysis of volatile compounds in Baijiu.

## Data Availability

No data were used to support this study.

## Supplementary Materials

Supplementary Materials Figure S1: three-dimensional GC-IMS spectrum. Table S1: peak area of volatile compounds in different sauce-flavor Baijiu. Table S2: ROAV of volatile compounds in different sauce-flavor Baijiu.
