# Supplementary material for: Insights into the Aroma Profile of Sauce-Flavor Baijiu by GC-IMS Combined with Multivariate Statistical Analysis
Source: J Anal Methods Chem. 2022 Mar 29;2022:4614330. doi: 10.1155/2022/4614330 (PMC8983223; doi:10.1155/2022/4614330)
Supplement: Supplementary Materials — Figure S1: three-dimensional GC-IMS spectrum. Table S1: peak area of volatile compounds in different sauce-flavor Baijiu. Table S2: ROAV of volatile compounds in different sauce-flavor Baijiu. [file 4614330.f1.zip › 4614330.f1/TableS1.pdf]

Code	Retention time (sec)	Retention index	Drift time	Compounds name by classes	Formula	Molecular weight
				Alcohols		
A1	329.268	941.9	1.14179	Ethanol	C2H6O	46.1
A2	488.48	1099.6	1.36917	2-Methyl-1-propanol	C4H10O	74.1
A3	576.353	1150	1.3812	Butanol	C4H10O	74.1
A4	679.915	1213.1	1.49569	3-Methyl-1-butanol	C5H12O	88.1
A5	916.397	1361.7	1.33174	1-Hexanol	C6H14O	102.2
				Total alcohols		
				Esters		
B1	306.03	904.5	1.33743	Ethyl acetate	C4H8O2	88.1
B2	343.864	963.6	1.45336	Ethyl propanoate	C5H10O2	102.1
B3	352.344	976.3	1.56115	Ethyl isobutyrate	C6H12O2	116.2
B4	361.207	989.5	1.47771	Propyl acetate	C5H10O2	102.1
B5	394.777	1021.5	1.61456	Isobutyl acetate	C6H12O2	116.2
B6	422.694	1045	1.56237	Ethyl butanoate	C6H12O2	116.2
B7	439.396	1059.1	1.65109	Ethyl 2-methylbutanoate	C7H14O2	130.2
B8	462.795	1078.8	1.65626	Ethyl 3-methylbutanoate	C7H14O2	130.2
B9	542.414	1130.5	1.74675	Isoamyl acetate	C7H14O2	130.2
B10	563.826	1142.8	1.68023	Ethyl pentanoate	C7H14O2	130.2
B11	720.397	1241.2	1.80137	Ethyl hexanoate	C8H16O2	144.2
B12	870.337	1335.6	1.91676	Ethyl heptanoate	C9H18O2	158.2
B13	897.495	1351	1.54144	Ethyl lactate	C5H10O3	118.1
B14	1091.142	1460.5	1.48427	Octanoic acid ethyl ester	C10H20O2	172.3
				Total esters		
				Acids		
C1	1193.741	1518.6	1.15362	Acetic acid	C2H4O2	60.1
C2	1314.769	1587	1.15924	Butanoic acid	C4H8O2	88.1
				Total acids		
				Ketones		
D1	267.682	816.6	1.11524	Acetone	C3H6O	58.1
D2	643.364	1188.5	1.63422	2-Heptanone	C7H14O	114.2
				Total ketones		
				Aldehydes		
E1	258.149	794.7	1.14431	Propanal	C3H6O	58.1
E2	263.564	807.1	1.28094	2-Methyl propanal	C4H8O	72.1
E3	275.338	834.1	1.06103	Acrolein	C3H4O	56.1
E4	317.65	924.6	1.4068	3-Methylbutanal	C5H10O	86.1
E5	364.962	995.1	1.42131	Pentanal	C5H10O	86.1
				Total aldehydes		
				Furans		
F1	1176.497	1508.8	1.09367	Furfural	C5H4O2	96.1
				Total Furans		

Peak area of volatile compound				
A	B	C	D	E
17661.15±1254.99	21590.66±2792.7	18343.26±1106.65	17471.56±2473.5	19639.45±2889.9
6186.09±567.33	6419.02±322.75	6213.38±651.87	6127.62±981.23	6148.99±398.98
4232.64±616.52	4150.49±420.11	4789.85±434.88	3367.4±659.72	3512.27±369.42
18353.82±1477.21	19766.43±564.94	18030.74±1226.6	16690.96±2116.67	18759.94±2243.14
1060.28±148.28	925.86±149.26	1082.35±136.95	1318.36±165.08	1020.91±143.86
47493.99±1548.99	52852.46±3582.14	48459.58±2831.43	44975.9±3051.8	49081.56±1978.83
23007.09±3307.56	23084.53±3684.94	22326.45±3517.61	26839.86±1464.94	25558.03±4642.06
11356.61±872.49	7744.6±1139.29	10259.57±897.84	10617.18±1462.95	7512.31±1210.27
9003.98±1130.7	7992.1±875.66	8421.96±662.26	8000.64±226.58	7984.86±1313.67
3162.67±654	2676.63±615.81	5211.49±867.44	6228.01±276.08	5326.79±624.45
991.79±208.88	1367.6±147.02	1506.94±138.69	1936.99±302.81	1833.26±372.24
19494.38±3279.91	21026.41±2497.26	20298.73±984.91	19737.59±3164.9	21181.99±2587.04
6227.99±632.48	3934.17±896.03	6865.56±1536.91	5269.83±97.94	4134.16±747.87
20644.94±3414.37	16350.63±3080.75	19515.13±2357.79	18607.94±382.83	17135.96±3351.75
4296.43±647.12	4472.85±257.21	4718.29±472.29	4285.19±387.93	5829.08±628.59
14645.14±1795.04	13269.99±1539.13	9014.33±2125.76	20476.17±2402.13	12815.93±597.56
23717.68±514.27	22959±1962.79	19432.08±4341.55	42144.88±3773.11	32281.24±4753.8
800.98±70.84	686.05±6.44	672.22±19.93	1592.24±139.25	906.45±121.1
6253.11±1163.79	9013.5±673.1	8340.45±584.91	7479.58±576.73	9405.82±699.79
1702.04±200.66	1053.75±87.93	1752.4±189.85	1311.97±87.21	1624.53±378.88
145304.83±2102.99	135631.8±8977.68	138335.62±4782.31	174528.07±1182.45	153530.41±5670.01
285.63±34.44	246.56±38.14	251.5±23.44	231.11±12.84	282.57±35.56
867.47±118.71	828.99±87.08	738.42±41.8	858.23±142.79	589±90.27
1153.11±120.41	1075.55±123.5	989.92±49.48	1089.34±138.95	871.56±121.78
5213.23±1236.09	4560.33±399.9	3853.88±673.84	5376.36±290.03	4452.98±550.45
593.64±92.9	355.16±45.01	531.84±76.67	633.5±78.56	533.6±18.51
5806.87±1143.44	4915.49±441.72	4385.72±710.71	6009.87±357.31	4986.58±566.26
2120.03±340.5	1669.24±248.6	2233.19±519.61	2422.2±521.09	2447.06±213.66
1273.89±299.77	1226.9±131.51	1553.93±186.4	1120.65±88.52	1374.93±181.67
941.93±89.27	923.07±13.3	814.23±148.35	746.59±91.65	848.25±130.93
928.39±130.86	747.3±96.45	802.75±99.96	516.2±97.63	597.85±55.69
872.01±129.67	714.09±107.29	895.41±153.71	1621.85±234.75	1408.92±79.81
6136.25±499.11	5280.6±317.66	6299.52±197.9	6427.49±694.39	6677.01±253.99
9343.4±950.27	8736.81±916.85	9541.12±359.7	10354.99±669.65	7657.02±942.84
9343.4±950.27	8736.81±916.85	9541.12±359.7	10354.99±669.65	7657.02±942.84

s in different sauce-flavor Baijiu

F	G	H	I	J
19270.57±3070.88	16852.95±2613.57	16785.82±1154.66	15995.72±2153.19	20459.96±1909.67
6933.01±747.93	6936.44±375.27	6755.24±681.89	6383.47±705	6650.45±622.06
4336.03±543.97	4232.46±221.55	3830.92±576.9	3533.67±366.64	3021.24±605.68
19526.23±734.01	17459.42±2647.82	18227.39±2945.37	16777.96±1397.11	18331.97±2933.39
1459.22±49.38	2197.8±219.97	1444.65±165.48	2919.35±489.91	1927.98±199.7
51525.06±2717.85	47679.07±4914.65	47044.02±2885.58	45610.17±2704.4	50391.6±4869.93
29224.09±2684.98	28292.07±4508.14	26447.3±3771.63	19714.28±3924.76	23065.96±2341.41
8829.33±1091.05	9431.76±731.95	10186.38±455.85	11849.54±1654.46	11154.89±2916.13
8719.53±962.76	8298.28±1430.17	7029.44±1011.69	6832.63±1633.29	6456.4±1100.26
4968.21±264.9	4701.38±314.29	3712.52±442.44	5650.7±422.75	5920.98±1391.81
1957.91±239	2485.75±170.32	728.53±100.05	1675.79±460.32	1188.41±305.36
22771.55±3678.42	20166.89±1962.39	27387.08±3359.21	22233.24±3849.72	25666.27±2459.29
5057.2±591.64	6827.02±847.12	4671.9±414.09	5657.13±428.52	5943.48±636.76
15923.21±2493.46	18524.29±4395.63	18365.49±1049.71	18383.3±2454.74	18548.67±711.88
6129.34±203.91	6151.98±1249.03	3724.99±273.41	4992.45±196.58	3419.8±594.59
14638.01±993.19	15903.67±1043.64	20796.53±3127.57	20836.45±4815.44	23022.64±2909.19
35652.38±1694.18	34646.99±815.12	36481.41±181.6	43859.96±4638.43	45822.84±5104.82
1299.66±101.54	1207.28±260.36	1765.28±218.87	3837.26±173.76	1796.38±227.53
9408.21±908.15	8854.77±531.1	6975.23±410.96	4712.41±569.18	5376.75±580.11
2086.34±455.82	1830.78±183.67	1336.44±197.31	3280±858.5	1584.25±202.91
166664.98±6096.88	167322.92±10349.32	169608.52±6029.7	173515.14±9002.86	178967.72±8750.36
295.66±52.23	314.26±42.03	262.15±12.15	224.29±24.21	226.49±31.03
937.66±176.78	780.28±30.85	497.73±50	1310.45±281.02	561.15±51.42
1233.32±223.92	1094.54±60.08	759.88±46.76	1534.74±303.32	787.64±21.99
4684.09±822.77	4622.32±478.57	4871.49±224.31	5602.41±167.06	3820.04±538.4
759.64±79.95	817.56±74.62	878.97±127.81	1608.58±375.96	580.35±9.76
5443.73±742.82	5439.87±404.08	5750.46±351.98	7210.99±421.81	4400.39±536.93
2685.59±391.91	2085.14±133.33	2386.18±341.02	3543.66±121.26	2576.86±241.72
1247.08±182.71	1114.24±130.45	1004.41±174.93	1363.98±283.21	1402.11±125.12
819.61±126.75	780.6±48.72	871.41±220.39	753.59±107.22	851.81±121.35
719.61±72.64	714.71±144.25	743.68±95.1	896.42±61.45	856.67±77.87
1480.78±229.82	1611.63±201.95	1263.14±165.3	1615.07±269.84	1420.04±130.12
6952.68±813.49	6306.31±196.96	6268.82±56.48	8172.73±494.86	7107.5±486.46
14384.64±1051.82	9530.39±1750.78	5130.73±601.75	11925.53±1155.31	6762.19±483.12
14384.64±1051.82	9530.39±1750.78	5130.73±601.75	11925.53±1155.31	6762.19±483.12
