# Supplementary material for: Insights into the Aroma Profile of Sauce-Flavor Baijiu by GC-IMS Combined with Multivariate Statistical Analysis
Source: J Anal Methods Chem. 2022 Mar 29;2022:4614330. doi: 10.1155/2022/4614330 (PMC8983223; doi:10.1155/2022/4614330)
Supplement: Supplementary Materials — Figure S1: three-dimensional GC-IMS spectrum. Table S1: peak area of volatile compounds in different sauce-flavor Baijiu. Table S2: ROAV of volatile compounds in different sauce-flavor Baijiu. [file 4614330.f1.zip › 4614330.f1/TableS2.pdf]

Compounds sorted by roav value	Odor threshold (ppm)
Ethyl hexanoate	55.33
Ethyl pentanoate	29.78
Ethyl 2-methylbutanoate	18
Ethyl butanoate	81.5
Ethyl 3-methylbutanoate	68.9
Ethyl isobutyrate	57.47
Octanoic acid ethyl ester	12.87
Acetone	62.08
Isoamyl acetate	93.93
3-Methylbutanal	16.51
Propanal	810
Propyl acetate	2700
Isobutyl acetate	922
Pentanal	725
Acrolein	519.75
Ethanol	11400
Butanol	2733.35
2-Methyl propanal	1300
Butanoic acid	964
Ethyl acetate	32551.6
Ethyl propanoate	19019.33
1-Hexanol	5370
Furfural	44029.73
3-Methyl-1-butanol	179190
Ethyl heptanoate	13153.17
Ethyl lactate	128083.8
2-Methyl-1-propanol	116161
2-Heptanone	42900
Acetic acid	160000

Odor Character	R[illegible]		
	A	B	C
sweet, fruity, pineapple, green banana	100±0	100±0	100±0
Apple	114.95±16.37	107.27±5.59	92.77±44.32
berry, pineapple, fruity	80.84±9.72	52.87±12.9	108.74±4.31
fruity, juicy fruit, pineapple, cognac	55.68±8.26	62.21±5.91	74.01±21.26
fruity, sweet apple, pineapple	70.01±12.49	57.7±13.93	82.26±11.68
sweet, ethereal, fruity, alcoholic, fusel, rummy	36.63±5.39	33.48±1.41	43.83±14.22
fruity, winey, sweet, apricot, banana, brandy, pear	30.91±4.35	19.73±0.21	40.81±14.23
sweet, fruity, etherous	19.65±5.03	17.73±1.37	18.29±4.81
banana, fresh	10.69±1.82	11.49±0.32	14.73±3.04
green, malty, cheesy	13.12±1.82	11.03±2.25	14.63±5.44
earthy, alcoholic, winey, whiskey, cocoa, nutty	0.61±0.09	0.5±0.08	0.78±0.01
sweet, ester	0.27±0.05	0.24±0.06	0.59±0.24
sweet, ester, medicinal	0.25±0.05	0.36±0.05	0.49±0.17
sickening, rancid, decayed	0.28±0.04	0.24±0.03	0.37±0.11
pungent	0.42±0.05	0.43±0.04	0.47±0.19
vinous, alcohol	0.36±0.02	0.46±0.09	0.48±0.15
sweet, malty, alcohol, medicinal	0.36±0.06	0.37±0.06	0.52±0.15
pungent	0.23±0.05	0.23±0.04	0.36±0.11
Cheesy, creamy	0.21±0.03	0.21±0.03	0.23±0.05
fruity, sweet, fingernail polish, etherous	0.16±0.02	0.17±0.02	0.2±0.05
sweet, fruity, rummy, juicy, grape, pineapple	0.14±0.01	0.1±0.01	0.16±0.05
green grass, plastic	0.05±0.01	0.04±0.01	0.06±0.02
bread, almond	0.05±0	0.05±0.01	0.06±0.01
sweet, malty, rancid, rubber,	0.02±0	0.03±0	0.03±0.01
fruity, pineapple, cognac, rummy, winey	0.01±0	0.01±0	0.02±0
sharp, tart, fruity, buttery, butterscotch	0.01±0	0.02±0	0.02±0
sweet, fusel, musty, alcohol, rubber, latex	0.01±0	0.01±0	0.02±0.01
sweet, mushroom	0±0	0±0	0±0
pungent, vinegar	0±0	0±0	0±0

OAV of volatile compounds in different sauce-flavor Baijiu						
D	E	F	G	H	I	J
100±0	100±0	100±0	100±0	100±0	100±0	100±0
91.02±15.87	74.46±7.11	76.51±8.01	85.33±6.36	105.97±16.43	88.32±19.86	94.24±17.53
38.67±3.97	40.27±10.76	43.82±7.05	60.69±8.73	39.38±3.68	39.96±5.53	40.48±8.29
32.14±7.19	44.67±1.9	43.65±9.13	39.5±3.55	50.96±6.16	34.53±6.22	38.57±7.55
35.64±3.16	42.48±3.86	36±6.42	42.98±10.55	40.43±2.45	34.02±6.88	32.84±4.53
18.35±1.23	23.78±1.2	23.64±3.58	23.03±3.61	18.56±2.75	15.18±4.51	13.73±3.26
13.45±1.46	21.47±2.4	25.37±6.71	22.73±2.46	15.75±2.32	32.48±10.07	15.09±3.22
11.39±0.49	12.38±1.6	11.77±2.44	11.89±1.18	11.9±0.49	11.49±1.47	7.45±0.91
6.04±0.97	10.89±2.61	10.15±0.81	10.47±2.18	6.01±0.42	6.76±0.75	4.41±0.77
4.14±1	6.34±1.36	6.75±0.37	6.92±1.43	6.83±0.9	6.93±1.14	6.35±1.21
0.4±0.11	0.52±0.09	0.52±0.1	0.41±0.02	0.45±0.06	0.56±0.07	0.39±0.04
0.3±0.02	0.35±0.08	0.29±0.03	0.28±0.02	0.21±0.02	0.26±0.01	0.27±0.08
0.28±0.06	0.35±0.12	0.33±0.03	0.43±0.04	0.12±0.02	0.23±0.07	0.16±0.05
0.3±0.06	0.34±0.04	0.32±0.06	0.36±0.05	0.26±0.03	0.28±0.02	0.24±0.03
0.19±0.03	0.28±0.05	0.25±0.05	0.24±0.01	0.25±0.07	0.18±0.03	0.2±0.02
0.2±0.04	0.3±0.01	0.26±0.03	0.24±0.03	0.22±0.02	0.18±0.02	0.22±0.04
0.16±0.02	0.22±0.05	0.25±0.04	0.25±0.02	0.21±0.03	0.17±0.03	0.13±0.03
0.11±0.01	0.18±0.01	0.15±0.03	0.14±0.01	0.12±0.02	0.13±0.03	0.13±0.02
0.12±0.03	0.11±0.03	0.15±0.02	0.13±0.01	0.08±0.01	0.17±0.04	0.07±0.01
0.11±0.01	0.14±0.04	0.14±0.01	0.14±0.02	0.12±0.02	0.08±0.01	0.09±0
0.07±0	0.07±0.02	0.07±0.01	0.08±0.01	0.08±0	0.08±0.02	0.07±0.02
0.03±0.01	0.03±0	0.04±0	0.07±0.01	0.04±0	0.07±0.02	0.04±0.01
0.03±0	0.03±0	0.05±0	0.03±0.01	0.02±0	0.03±0	0.02±0
0.01±0	0.02±0	0.02±0	0.02±0	0.02±0	0.01±0	0.01±0
0.02±0	0.01±0	0.02±0	0.01±0	0.02±0	0.04±0	0.02±0
0.01±0	0.01±0	0.01±0	0.01±0	0.01±0	0±0	0.01±0
0.01±0	0.01±0	0.01±0	0.01±0	0.01±0	0.01±0	0.01±0
0±0	0±0	0±0	0±0	0±0	0±0	0±0
0±0	0±0	0±0	0±0	0±0	0±0	0±0

Average
100
93.08
54.57
47.59
47.44
25.02
23.78
13.39
9.16
8.3
0.51
0.31
0.3
0.3
0.29
0.29
0.26
0.18
0.15
0.13
0.09
0.05
0.04
0.02
0.02
0.01
0.01
0
0
