# Supplementary figures and images for: Insights into the Aroma Profile of Sauce-Flavor Baijiu by GC-IMS Combined with Multivariate Statistical Analysis
Source: J Anal Methods Chem. 2022 Mar 29;2022:4614330. doi: 10.1155/2022/4614330 (PMC8983223; doi:10.1155/2022/4614330)

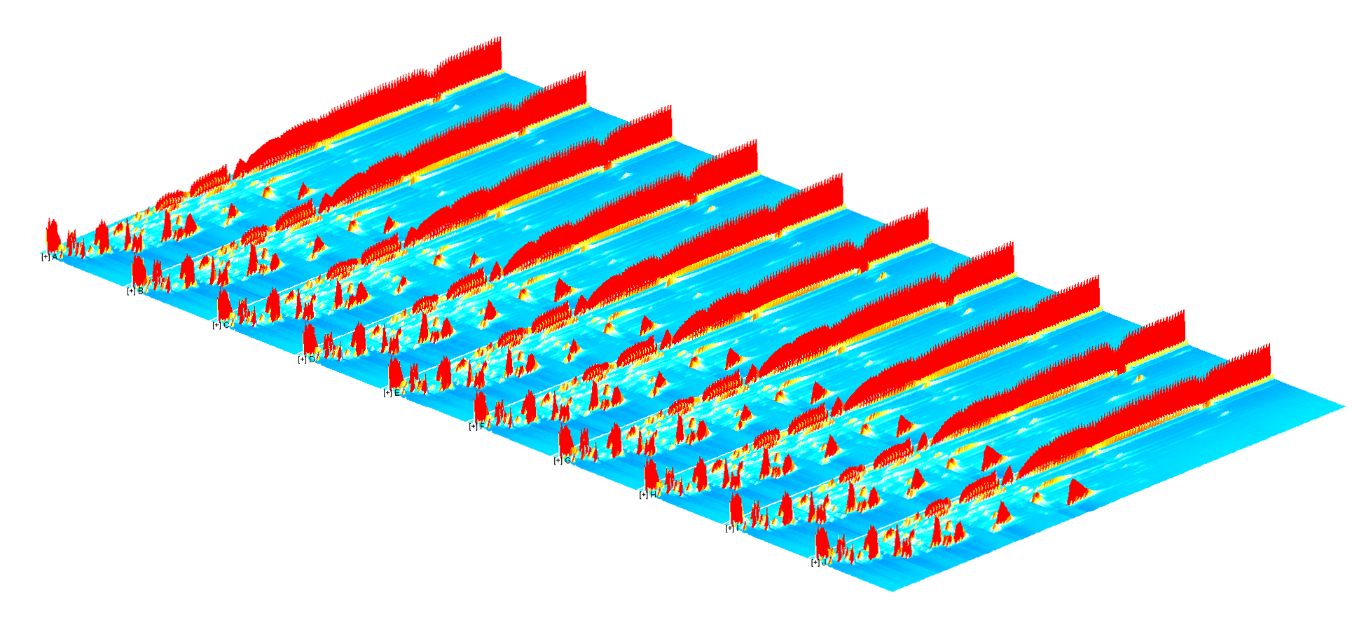


**Figure S1.** Three-dimensional GC-IMS spectrum.

Supplement: Supplementary Materials — Figure S1: three-dimensional GC-IMS spectrum. Table S1: peak area of volatile compounds in different sauce-flavor Baijiu. Table S2: ROAV of volatile compounds in different sauce-flavor Baijiu. [file 4614330.f1.zip › 4614330.f1/figure s1.docx]
